# OIL: A Closed-Loop Periodic Batch Adaptation Framework for Object Detection on Industrial Variable-Speed Conveyor Lines

**DOI:** 10.3390/s26175423

**Published:** 2026-08-27

**Authors:** Xiang Liu, Jing Fang, Haiqiao Liu, Zichao Gong, Zhiling Peng, Jing Dong

**Affiliations:** 1 School of Electrical and Information Engineering, Hunan Institute of Engineering, Xiangtan 410014, China; liuxiang5741@stu.hnie.edu.cn (X.L.); zichao_gong@163.com (Z.G.); pengzhiling@stu.hnie.edu.cn (Z.P.); 2 Hunan Provincial Third Surveying and Mapping Institute, Changsha 410008, China; itxjf3@gmail.com; 3 Aerospace Technology Research Institute, Central South University, Changsha 410008, China; dongjing@csu.edu.cn

**Keywords:** variable-speed conveyor line, periodic batch adaptation, object detection, motion blur, detection accuracy

## Abstract

To address motion blur and the degradation of object detection performance caused by variations in industrial conveyor-belt speed, this paper proposes OIL, a closed-loop periodic batch adaptation framework. First, the framework employs a YOLOv8 detector incorporating the Global Attention Mechanism (GAM) and uses the Classification Index Weight (CIW), which consists of detection confidence and the temporal consistency of predicted bounding boxes between adjacent frames. Second, the detection results are divided into three intervals according to the CIW and its constituent metrics: interval A, with a CIW below 0.3; interval B, with a CIW between 0.3 and 0.5; and interval C, with a CIW above 0.5. Results in interval A are treated as negative samples, those in interval B as ambiguous samples, and those in interval C as positive samples. Positive samples are used to train the candidate model, ambiguous samples are stored in a buffer and re-evaluated in subsequent cycles, and negative samples are excluded from the current model update. Finally, when the accumulated number of positive samples reaches a predefined threshold, the system trains a candidate model using the baseline data and newly collected samples. The candidate model is then compared with the currently deployed model on a fixed manually annotated evaluation set. The candidate model is deployed only when its overall performance improves; otherwise, a rollback is performed. Experiments on a coal conveyor line demonstrate that, at 25× speed, incorporating OIL into YOLOv8 + GAM improves Recall by 34.3 percentage points compared with the same detector without OIL. In the variable-speed experiments ranging from 5× to 25×, Recall remained above 64.3%, indicating that the proposed framework can effectively suppress missed detections under high-speed operating conditions.

## 1. Introduction

Industrial production lines provide an important foundation for improving manufacturing efficiency and product quality [1]. However, conventional production lines still face problems such as fluctuations in product quality, limited production efficiency, high labor costs, and occupational safety risks. In recent years, the rapid development of computer vision technology has made object detection and classification important supporting technologies for production-line automation. These technologies can accurately locate and identify objects in images or videos, thereby improving production efficiency and operational reliability [2]. Deep learning detectors such as YOLO and Faster R-CNN have achieved high detection accuracy while maintaining real-time performance [3,4,5,6,7]. In production-line scenarios, these methods can be used to automatically perform complex inspection tasks, achieve real-time condition monitoring, reduce human error, and provide effective feedback for production-process optimization [8]. Therefore, object detection and classification technologies provide important technical support for the development of intelligent manufacturing.

Despite significant progress, object detection in industrial scenarios still faces numerous challenges. First, existing models have limited generalization capabilities and struggle to adapt to dynamic changes in product types and operating conditions. They therefore generally require frequent parameter tuning or retraining, increasing system maintenance and operating costs [9]. Second, variations in conveyor-belt speed can readily cause motion blur, weakening key image features such as edges and textures and consequently increasing the risks of false and missed detections [10,11,12,13,14]. In addition, industrial datasets commonly suffer from class imbalance, with abundant normal samples but relatively few defective or other rare samples [15]. High annotation costs and inconsistent labels further increase the difficulty of iterative model training. Meanwhile, factors such as camera shake, belt vibration, uneven illumination, abrupt changes in operating conditions, and limited hardware resources may reduce the robustness of detection systems [16]. Together, these factors constrain the level of automation and long-term operational reliability of industrial detection systems.

Extensive research has been conducted worldwide on object detection for production lines [17]. Sun et al. [18] proposed LDD-Net for multiscale small-defect detection. Their method divides high-resolution inspection images into multiple units, thereby reducing the effects of uneven illumination and image skew. Zhao et al. [19] proposed combining contrastive learning, transfer learning, and attention mechanisms to detect small components during assembly. They reduced the dependence on annotated data through an unsupervised pretrained encoder and multiscale feature fusion. Yang et al. [20] proposed MemSeg, which integrates self-supervised learning, memory pooling, and multiscale feature fusion into an end-to-end segmentation network, achieving both detection accuracy and inference efficiency in semi-supervised industrial defect detection. Kong et al. [21] proposed AMBi-GAN, which integrates a bidirectional long short-term memory network with an attention mechanism. Through adversarial training between the generator and discriminator, this method improved multidimensional time-series anomaly detection in the Industrial Internet of Things under conditions of limited annotated samples.

To mitigate the performance degradation caused by image blur, researchers have proposed various attention mechanisms to enhance the extraction of fine-grained texture features [22,23,24]. Although high-speed cameras can alleviate motion blur by shortening the exposure time, they cannot eliminate it. Consequently, algorithmic deblurring and image enhancement methods for images involving nonuniform motion have received considerable attention. Guo et al. [25] investigated motion blur in visual tracking, constructed the BVT benchmark dataset containing samples with different degrees of blur, and proposed an adaptive deblurring method based on a generative adversarial network (GAN). Ma et al. [26] proposed combining attention-based dehazing, GAN-based deblurring, and the Pipe-Yolact-Edge segmentation network for sewer-pipeline defect detection. Although these methods require no additional imaging equipment, they generally introduce additional preprocessing overhead, making it difficult to meet the requirements of real-time online detection. Furthermore, image details cannot be reliably recovered from severely degraded images.

In recent years, adaptive learning strategies such as online learning, continual learning, domain adaptation, and pseudo-label-based self-training have attracted considerable attention. These methods can use newly collected data to mitigate performance degradation caused by changes in data distribution. However, most existing methods focus on general domain-shift problems and typically depend on sufficient target-domain data, predefined adaptation strategies, or additional optimization procedures. For industrial production lines, changes in data distribution are often caused by physical factors such as variations in conveyor speed, motion blur, illumination fluctuations, and limited available data. Therefore, an efficient online adaptation framework capable of automatically evaluating newly collected samples and dynamically updating the detection model remains necessary.

Although newer YOLO models have continuously improved the accuracy–efficiency trade-off in real-time object detection, YOLOv8 remains suitable as the base model for lightweight industrial detection tasks because of its stable detection performance, moderate computational complexity, mature implementation, and ease of deployment. Previous studies [27,28] have also demonstrated the potential of YOLOv8 for industrial surface-defect detection. Therefore, this study selects YOLOv8n as the base detector to validate the proposed CIW-based closed-loop periodic batch adaptation mechanism under the resource constraints of an industrial control computer. This selection is based primarily on engineering applicability and deployment reliability and does not imply that YOLOv8n outperforms subsequent YOLO models.

To address motion blur and detection performance degradation caused by variations in conveyor-belt speed, this paper proposes OIL, a closed-loop periodic batch adaptation framework for object detection on industrial variable-speed conveyor lines. The main contributions of this study are as follows:

1. A closed-loop periodic batch adaptation framework is proposed for industrial variable-speed conveyor-line detection. The framework accepts or rejects candidate models according to a comprehensive performance evaluation metric and constructs a closed-loop update strategy with a rollback mechanism. It can use a small amount of newly collected data to adapt to changes in operating conditions, thereby improving the robustness of the detection system in complex industrial environments.
2. The Global Attention Mechanism (GAM) is introduced into the YOLOv8 backbone. By jointly modeling channel and spatial attention, GAM integrates local features of coal fragments, surrounding contextual information, and global semantic information, thereby enhancing the model’s feature representation capability under dynamic operating conditions.
3. A three-level sample classification strategy based on the Classification Index Weight (CIW) is proposed. This strategy divides newly collected samples into positive, ambiguous, and negative samples, extending the conventional binary classification of positive and negative samples. It preserves useful information contained in uncertain samples while reducing the influence of unreliable pseudo-labels on model training.

As shown in Table 1, the proposed OIL method is compared with representative existing methods in terms of the detection framework, attention mechanism, sample classification strategy, data efficiency, variable-speed adaptability, and model update mechanism.

The remainder of this paper is organized as follows. Section 1 presents the introduction. Section 2 describes the proposed OIL method in detail. Section 3 presents the experimental setup, results, and discussion and validates the effectiveness of the proposed method through ablation and comparative experiments. Section 4 concludes the paper and outlines directions for future work.

## 2. OIL Method

### 2.1. Three-Stage Closed-Loop Self-Optimizing Learning Framework

**Figure 1:**
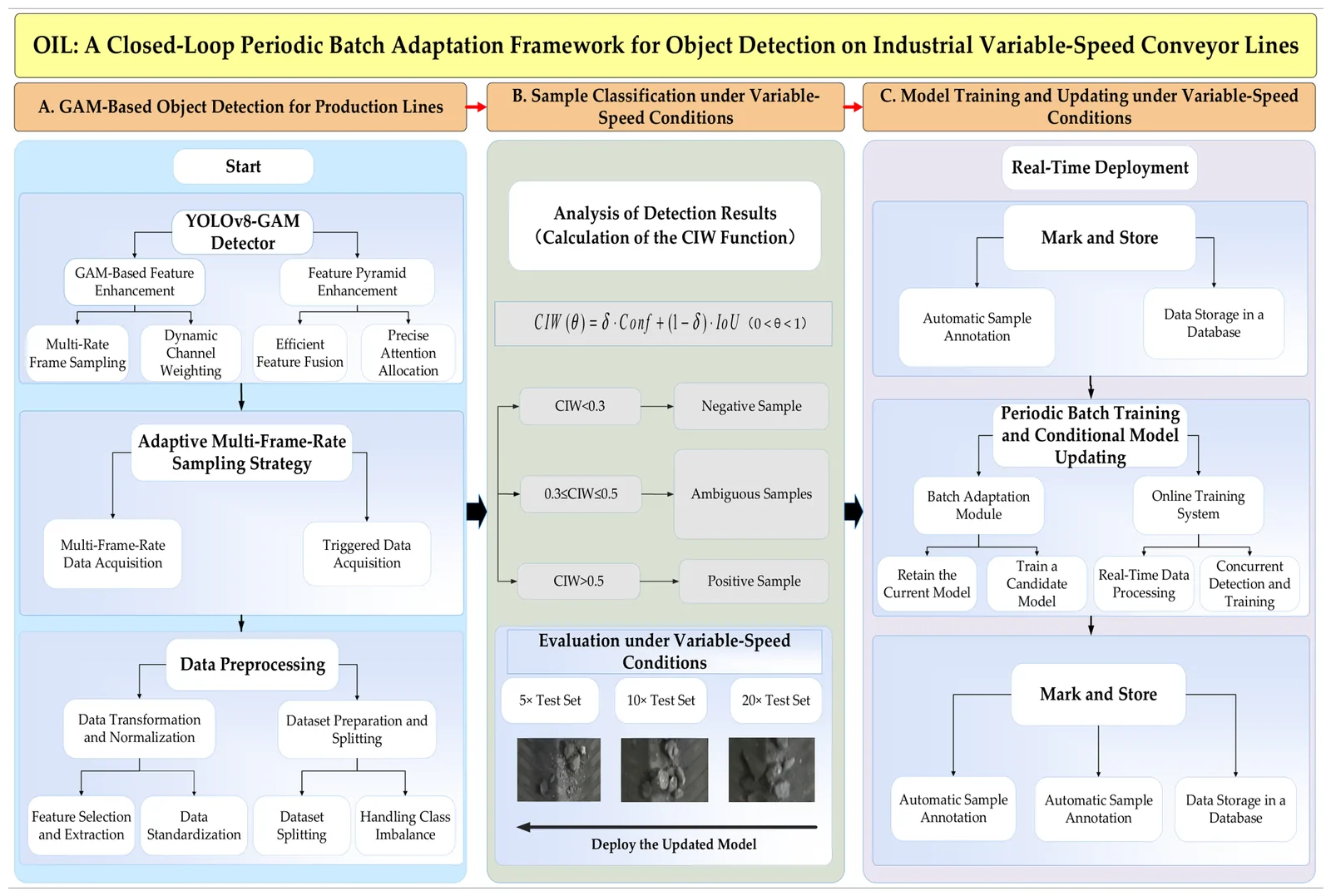
Overall architecture of the OIL method.

To achieve high-accuracy object detection in variable-speed industrial production-line scenarios, this paper proposes a closed-loop periodic batch adaptation framework, termed OIL. “Online” means that the system continuously collects, processes, and manages data from new operating conditions during deployment on an actual production line, whereas “batch” means that newly acquired samples that have passed reliability screening are progressively incorporated into the model adaptation process. It should be emphasized that OIL does not update model parameters immediately on a frame-by-frame or sample-by-sample basis. Instead, periodic batch training is triggered when the accumulated number of online samples reaches a predefined threshold, and the model performance evaluation results are used to determine whether the deployed model should be updated. Therefore, the proposed method constitutes a closed-loop periodic batch adaptation mechanism for practical industrial scenarios. Its overall workflow is illustrated in Figure 1.

First, in the sample classification module, the system performs three-level sample classification according to the CIW. Samples with a CIW below 0.3 are regarded as negative samples. Samples with a CIW > 0.5 are regarded as positive samples and can be directly used for batch learning. Samples with 0.3 ≤ CIW ≤ 0.5 are regarded as ambiguous samples. These samples are not directly used for training in the current cycle but are stored as buffered samples. After one batch-training cycle has been completed and the model has been updated, the new model is used to redetect and re-evaluate the buffered samples. If the new CIW increases to above 0.5, the corresponding sample is converted into a positive sample and included in the next training cycle. If the CIW remains below 0.5, the sample is discarded. This strategy preserves useful information from ambiguous samples while reducing the interference of low-quality pseudo-labels with model training.

Second, in the object detection algorithm, an improved YOLOv8 model integrated with the Global Attention Mechanism (GAM) was adopted for feature learning and target detection in conveyor-belt images. Through multiscale spatial perception, dynamic channel weighting, efficient feature fusion, and precise attention allocation, GAM enhances the model’s representation capability for coal targets and complex background features. Meanwhile, the system acquires conveyor-belt images at a fixed frame rate and directly feeds the acquired image stream into the detection module, providing data support for subsequent detection and model training.

Finally, in the model training and updating module, the screened samples are automatically annotated, stored in a database, and used for batch learning and model updating. The system evaluates the performance of the newly trained model based on real-time detection feedback. When the overall performance of the candidate model is superior to that of the currently deployed model, the system updates and deploys the new model; otherwise, the original model is retained. The updated model is then reintroduced into the data acquisition and detection stages for self-optimization, ensuring that the model maintains stable detection performance while adapting to changes in the environment and object characteristics. This process continuously improves object detection performance and ultimately forms a complete closed-loop feedback system. The proposed method can continuously adapt to motion blur and changes in operating conditions caused by variations in conveyor speed, thereby improving the stability and robustness of object detection on production lines.

### 2.2. Sample Classification Method Under Variable-Speed Conditions

The quality of pseudo-labeling is crucial to closed-loop periodic batch adaptation. This study evaluates detection results using confidence Conf, intersection over union IoU, and the Classification Index Weight CIW obtained by combining these two metrics [29,30,31]. Online images do not contain manually annotated ground-truth bounding boxes. Therefore, the IoU used in this study is not calculated between a predicted bounding box and a ground-truth bounding box; instead, it represents the temporal-consistency IoU between a predicted bounding box in the current frame and its matched predicted bounding box in the previous frame. Conf reflects the reliability of the class prediction, whereas temporal IoU reflects the stability of object localization across consecutive frames. Together, these metrics are used for pseudo-label screening and sample classification. This enables the system to adapt to changes in environmental and object characteristics while maintaining the continuity of online detection.

During online pseudo-label selection, the detection confidence Conf is obtained from the deployed YOLOv8 detector. For the i-th candidate bounding box, its confidence is defined as the maximum predicted class probability of that bounding box across all classes:

(1) $$Conf_{i}=\max P_{i,c}\;(c\in C)$$

Among these variables, $P_{i,c}$ denotes the predicted probability that candidate bounding box i belongs to a class, and C denotes the set of classes. Because the task considered in this study is single-class coal detection, $Conf_{i}$ is the predicted probability that the candidate bounding box belongs to the coal class. This score represents the reliability of the model’s object-class prediction.

The newly collected online images do not contain manually annotated ground-truth bounding boxes. Therefore, the IoU used in this study is not calculated between predicted bounding boxes and ground-truth annotations, but is defined as the temporal localization consistency between matched predicted bounding boxes in adjacent frames. For a candidate bounding box in the current frame t, its corresponding bounding box in the previous frame t−1 is selected according to class consistency and the maximum-overlap criterion. Because this study focuses on single-class coal detection, class consistency is naturally satisfied for coal predictions, and the matching is mainly determined by the largest overlap between adjacent-frame predictions. When multiple coal objects are close to one another, the previous-frame prediction with the largest IoU is selected as the temporal match. If a new object enters the frame, or if no corresponding prediction exists in the previous frame, the temporal IoU is set to zero, and the corresponding sample is conservatively evaluated in the CIW-based screening process. The temporal IoU is then defined as follows:

(2) $$IOU_{i}^{temp}=\frac{\left|b_{i}^{t}\cap b_{j}^{t-1}\right|}{\left|b_{i}^{t}\cup b_{j}^{t-1}\right|}$$

Here, ∣⋅∣ denotes the area of a bounding box. A higher temporal IoU indicates that the predicted object location is more stable across consecutive frames and that the corresponding pseudo-label has higher localization reliability. Conversely, a lower temporal IoU may indicate motion blur, detection instability, false detection, or the absence of a reliable temporal correspondence. It should be noted that temporal IoU is affected by conveyor speed, camera frame rate, and inter-frame object displacement. Under high-speed conditions or low frame rates, a correctly detected object may still obtain a relatively low temporal IoU because it moves substantially between two adjacent frames. Therefore, the temporal IoU in this study is not used as a ground-truth localization metric. Instead, it serves only as a temporal-stability indicator for pseudo-label screening and is combined with the confidence output by YOLOv8 to calculate the CIW and reduce the influence of unreliable pseudo-labels.

During online batch adaptation, the CIW is responsible for online sample screening, and newly collected samples are classified as positive, ambiguous, or negative samples. A candidate prediction is classified as a positive sample and used as a pseudo-label for online batch training only when the detection confidence satisfies Conf>0.5, and the IoU satisfies IoU>0.5. If either Conf or IoU is below 0.3, the prediction is regarded as a negative sample and is excluded from the current model update. All remaining candidate predictions are classified as ambiguous samples. These samples are temporarily excluded from the current batch-training cycle and retained for subsequent re-evaluation to prevent uncertain pseudo-labels from interfering with model updating. This criterion effectively excludes high-confidence false detections with unstable localization and unreliable predictions severely affected by motion blur. This study further constructs the CIW to jointly represent class-prediction reliability and temporal localization stability. The CIW ranges from 0 to 1, with a higher value indicating a more reliable pseudo-label. Its measurement function θ is defined as follows:

(3) θ=δ⋅Conf+(1−δ)⋅IoU

The CIW score measures the overall reliability of a candidate sample and is used to complete sample classification. In this study, the weighting coefficient δ is set to 0.5, assigning equal importance to the detection confidence Conf and the IoU in the CIW calculation. The former reflects the reliability of the model’s object-class prediction, whereas the latter reflects the spatial localization stability of the predicted bounding box across adjacent frames. Based on this setting, the sample classification rules are defined as follows:

(4) $$Sample\;Type\;f(x)=\left\{\begin{array}{ll} Negative\;Sample & \mathrm{if}\theta <\theta_{low}\\ Ambiguous\;Sample & \mathrm{if}\theta_{low}\leq \theta \leq \theta_{high}\\ Positive\;Sample & \mathrm{if}\theta >\theta_{high} \end{array}\right.$$

In the above equation, *θ*_low_ and *θ*_high_ denote the lower and upper classification thresholds, respectively. In this study, *θ*_low_ = 0.3 and *θ*_high_ = 0.5. The detailed classification rules are presented in Table 2.

### 2.3. GAM-Based YOLOv8 Network

**Figure 2:**
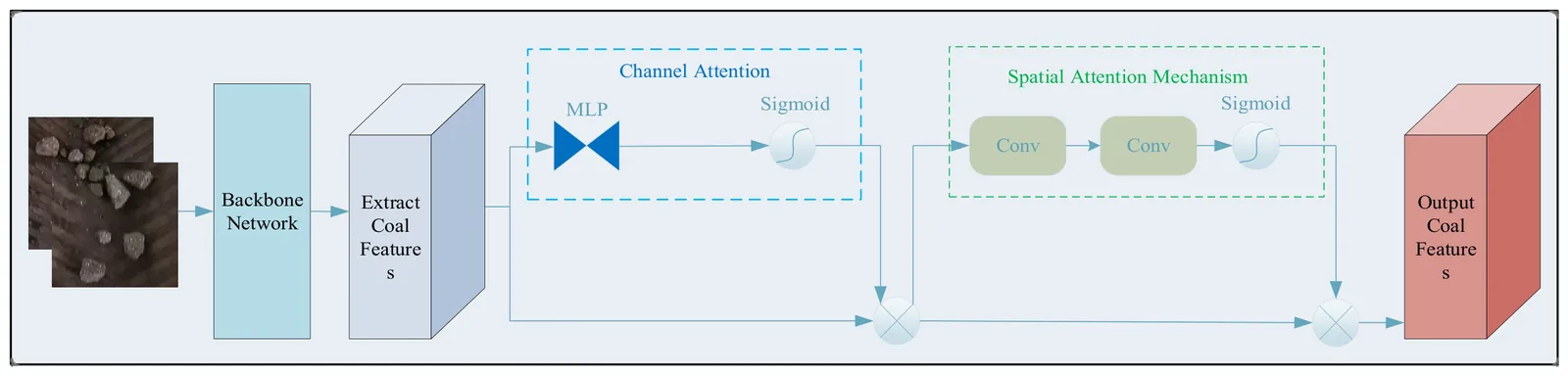
Channel and spatial attention modules of GAM.

The core of GAM is to preserve global information through dual-channel and spatial attention modules, thereby avoiding information loss caused by dimensional separation [32]. The channel attention module of GAM maintains information integrity through three-dimensional permutation and employs a two-layer multilayer perceptron (MLP) to strengthen cross-dimensional correlations. Global average pooling is then used to compress the feature map into channel-importance weights, which are adjusted through fully connected layers and activation functions and subsequently multiplied by the original feature map to enhance informative channel features and suppress irrelevant ones, as expressed in Equations (1) and (2). The spatial attention submodule uses two convolutional layers to integrate spatial features and omits max pooling to preserve detailed information. It also applies a reduction ratio and takes the feature map processed by the channel attention module as its input. Spatial features are extracted through convolution to generate attention weights, which are multiplied by the feature map to highlight important regions and suppress the background. GAM combines channel and spatial attention by first enhancing the features through channel attention and then using spatial attention to focus on target regions. This process enables global information perception and the capture of long-range dependencies, thereby improving the model’s ability to exploit contextual information. The detailed structure is shown in Figure 2.

(5) $$F_{2}=M_{C}(F_{1})⊗F_{1}$$

(6) $$F_{3}=M_{C}(F_{2})⊗F_{2}$$

In the above equations, the M_C_ module adjusts the channel-correlation weights of the feature map. During this process, the input feature F_1_ is processed by the M_C_ module and multiplied by the original feature to obtain the enhanced feature F_2_. Similarly, the same operation is applied to F_2_ to generate F_3_, progressively optimizing feature representation and improving the model’s ability to capture critical information in complex tasks.

**Figure 3:**
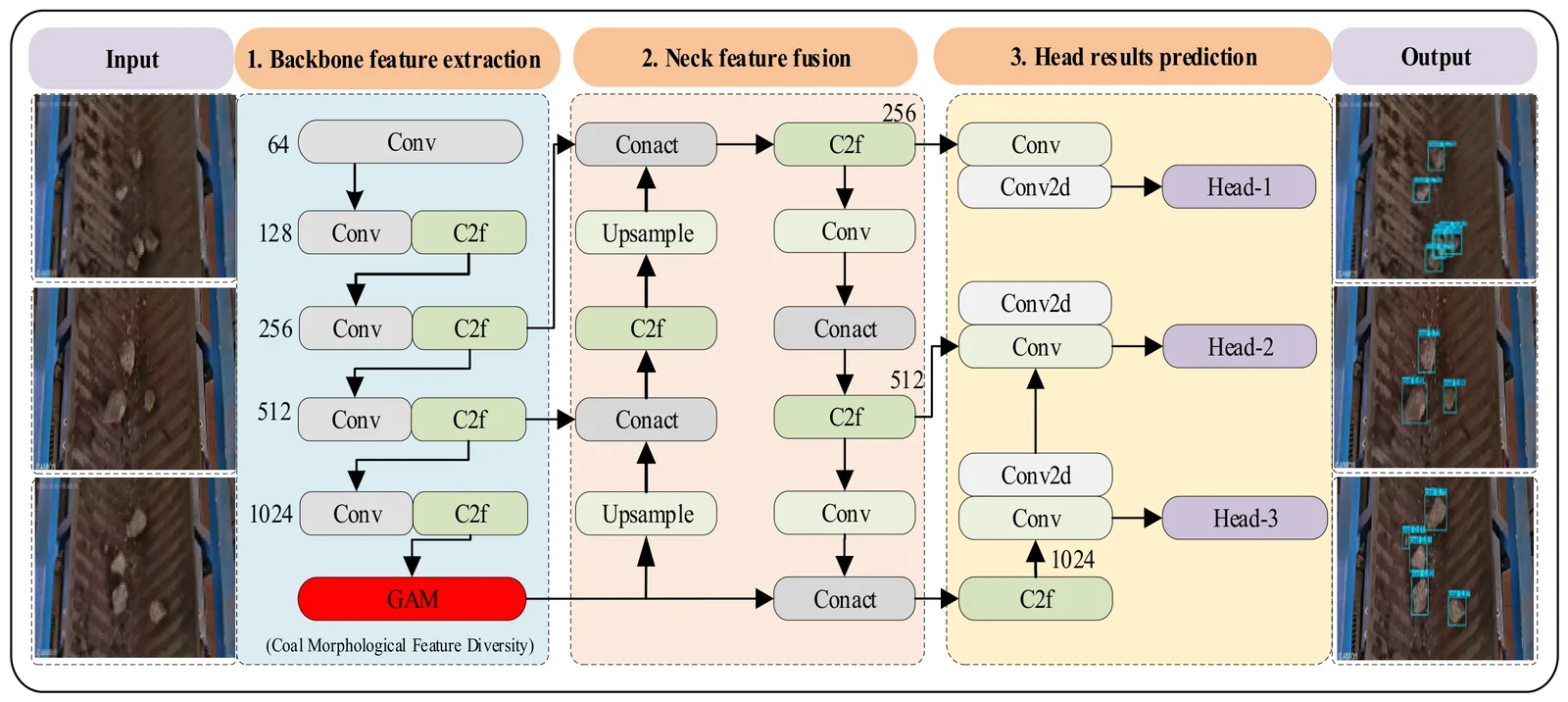
Overall structure of the YOLOv8 + GAM method.

To address the insufficient preservation of information across the channel and spatial dimensions by conventional attention mechanisms, this study improves the YOLOv8 network architecture [33]. The network mainly consists of three components: the Backbone, Neck, and Head. GAM (Global Attention Mechanism) is introduced into the Backbone and combines channel attention with spatial attention. When the model encounters occlusion, illumination variations, noise interference, or diverse object poses, GAM can more effectively utilize global information to suppress interfering factors. For small-object detection, small objects occupy limited spatial regions in an image, and their principal information is therefore represented mainly in the channel dimension. The channel attention mechanism of GAM can enhance channel features associated with small objects while suppressing interference from irrelevant channels, thereby improving small-object detection accuracy. After GAM is integrated into the backbone, the network can more effectively understand critical image information while reducing computational complexity. The resulting network structure is shown in Figure 3.

The model is trained using the YOLOv8 loss function. It should be noted that the Classification Index Weight (CIW) does not participate in YOLOv8 backpropagation and is not used as a weighting coefficient for the classification loss, bounding-box regression loss, or total detection loss. The CIW is used only during the online stage to evaluate the reliability of newly collected samples and classify them as positive, ambiguous, or negative samples.

Positive samples selected using the CIW are trained using the standard YOLOv8 detection loss. The total detection loss consists of the classification loss, the Complete IoU (CIoU)-based bounding-box regression loss, and the Distribution Focal Loss (DFL), and is expressed as follows:

(7) $$L_{\det}=\lambda_{cls}L_{cls}+\lambda_{box}L_{CIOU}+\lambda_{dfl}L_{DFL}$$

where $\lambda_{cls}$, $\lambda_{box}$, $\lambda_{dfl}$ denote the weighting coefficients for the classification loss, bounding-box regression loss, and *L_DFL_*, respectively. The default YOLOv8 implementation is adopted to further optimize bounding-box regression accuracy.

Binary cross-entropy loss is used as the classification loss to optimize the model’s ability to predict the coal class. It is defined as follows:

(8) $$L_{cls}=-\sum_{i=1}^{N}\left[y_{i}\log\left(\partial \left(z_{i}\right)\right)+\left(1-y_{i}\right)\log\left(1-\partial \left(z_{i}\right)\right)\right]$$

where $Z_{i}$ denotes the classification prediction output by the detection head, ∂ denotes the sigmoid activation function, and $y_{i}$ denotes the corresponding class-supervision label. For positive samples, $y_{i}$ is the coal-class label. For negative-sample images, the annotation is empty because they contain no coal objects, and these samples are primarily used to enhance the model’s ability to distinguish background regions.

The CIoU loss is used for bounding-box regression to simultaneously account for the overlap between the predicted and training-label bounding boxes, the distance between their center points, and the consistency of their aspect ratios. It is defined as follows:

(9) $$L_{CIOU}=1-CIOU$$

### 2.4. Model Training and Updating Strategy Under Variable-Speed Conditions

**Figure 4:**
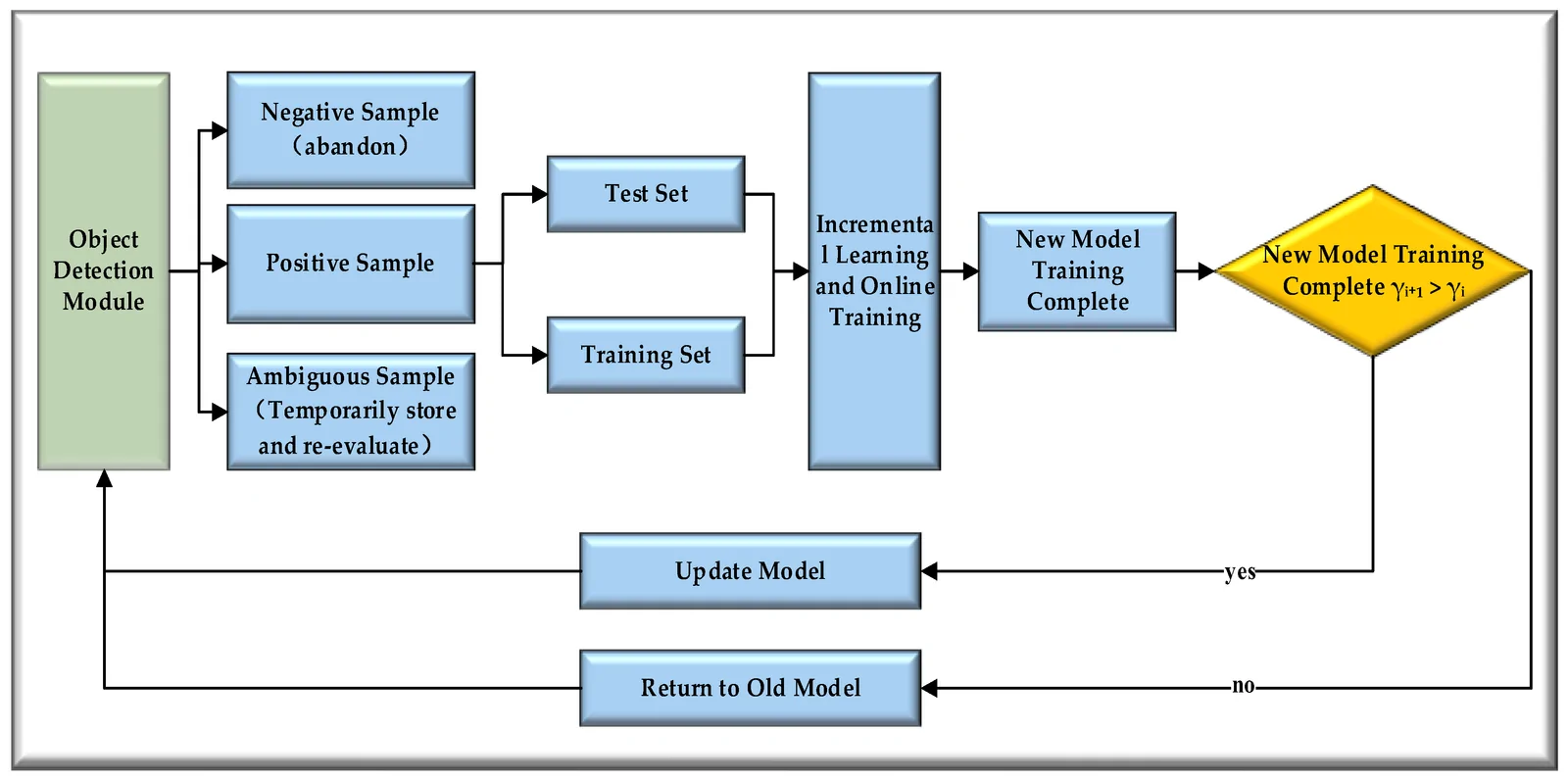
Dynamic iteration and optimization flow of the object detection model.

To achieve the continuous optimization and dynamic updating of the object detection model, this study constructs a model iteration process based on a closed-loop periodic batch adaptation mechanism. First, the object detection module screens the newly collected data for reliability and classifies them into positive, negative, and ambiguous samples. Ambiguous samples are not used as training labels in the current model update, thereby preventing unreliable pseudo-labels from interfering with the training process. Only the positive samples that pass the screening process are retained as pseudo-labeled data. Negative samples are excluded from the current training cycle and do not participate in online batch training. The positive samples are then further divided into training and internal monitoring sets and incorporated into the batch adaptation module to generate a new candidate model. After candidate-model training is completed, the system tests the candidate model using a predefined comprehensive performance evaluation metric and compares it with the currently deployed model. When the comprehensive evaluation result of the candidate model is superior to that of the current model, the system updates the model parameters and deploys the new model to the object detection module. Otherwise, the current update is rejected, and the original model is retained. Through continuous data iteration, this process enables the model to incorporate new samples while maintaining stable online detection. The entire process forms a closed-loop control system comprising sample screening, batch training, performance evaluation, and conditional updating. The detailed workflow is illustrated in Figure 4.

Within the iterative optimization framework of the object detection system, the Recall and mean average precision at an IoU threshold of 0.5 (mAP50) are used to construct a γ function for a more rigorous evaluation of the performance of the new model. Recall measures the model’s ability to identify all positive samples. A higher Recall indicates a lower missed-detection rate for positive samples. The mAP50 metric is used to evaluate the accuracy of coal bounding-box detection and provides a comprehensive assessment of the model’s overall detection performance. A higher mAP50 indicates a better balance between detection precision and recall across the object classes. The γ function is a comprehensive metric that combines Recall and mAP50, thereby overcoming the limitations of using a single metric. The γ function is therefore used to evaluate the performance of the new model and is defined as follows:

(10) $${\mathrm{mAP}}_{i}50=\frac{1}{N}\sum_{i=1}^{N}AP_{i}$$

(11) $$Recall=\frac{TP}{FN+TP}\;$$

(12) γi=αRecalli+(1−α)mAPi

In Equation (12), α is set to 0.5, indicating that mAP50 and Recall are assigned equal weights because the proposed method considers both localization accuracy and missed-detection suppression. Increasing α increases the weight assigned to Recall, whereas decreasing α increases the weight assigned to mAP50. This setting provides a balanced evaluation criterion for model update decisions. *N* denotes the total number of object classes in the detection task, $AP_{i}$ denotes the average precision of the *i*-th class, TP (true positive) denotes the number of correctly detected coal objects, and FN (false negative) denotes the number of coal objects that are present but not detected.

This study proposes a combined allocation method for dividing the samples. A baseline dataset was collected and constructed under the 5× conveyor-speed condition. It contains 800 training images, 200 test images, and 100 validation images, for a total of 1100 images. All images in the dataset were manually annotated and include clear coal samples, blurred coal samples, and coal-free background samples. The model trained on the baseline dataset is referred to as the $M_{0}$ baseline model. The composition of the 5× baseline dataset is presented in Table 3.

During actual deployment, the $M_{0}$ model is deployed to perform real-time detection on continuous images newly collected under new operating conditions. The system classifies the online samples into positive, ambiguous, and negative samples according to the CIW. The pseudo-labels of the positive samples are stored in the online sample pool. Ambiguous samples are temporarily stored and re-evaluated using the updated model during subsequent detection cycles. Negative samples are excluded from the current training cycle and do not participate in online batch training, thereby preventing difficult coal objects from being incorrectly labeled as background.

The model is not updated immediately after each image frame is detected. Instead, one batch-training cycle is triggered after 300 positive samples have accumulated in the online sample pool. Specifically, 200 images are randomly selected from this batch of positive samples and combined with the 800 images in the baseline training set to form the batch-training set for the current cycle. The remaining 100 pseudo-labeled online images are combined with the 200 manually annotated baseline test images to form an internal batch monitoring set. This set is used only to monitor the behavior of the candidate model during the current batch-adaptation cycle. It is not regarded as an independent test set and is not used for final performance reporting or model-update decisions. The resulting batch dataset is presented in Table 4.

Meanwhile, to prevent variations in the evaluation data from interfering with model update decisions, a fixed and independent model-update evaluation set is constructed. This evaluation set covers five conveyor-speed conditions, 5×, 10×, 15×, 20×, and 25×, and contains manually annotated clear coal images, blurred coal images, and manually verified coal-free background images. The evaluation set does not participate in baseline-model training, online sample screening, or candidate-model training and does not overlap with the test sets under the different speed conditions. It is used only to compare the comprehensive performance of the currently deployed model $M_{0}$ and the candidate model under identical data conditions and to provide a consistent basis for model update or rollback decisions. The detailed composition of the evaluation set is presented in Table 5.

Let the currently deployed model be $M_{i}$, with a comprehensive evaluation metric of $\gamma_{i}$ on the fixed model-update evaluation set, and let the candidate model be $M_{i+1}^{c}$, with a comprehensive evaluation metric of $\gamma_{i+1}^{c}$. When the candidate model satisfies

(13) $$\gamma_{i+1}^{c}>\gamma_{i}$$

the candidate model is considered to have superior overall detection performance. The current update is therefore accepted, and the candidate model is deployed as the online detection model for the next cycle:

(14) $$M_{i+1}=M_{i+1}^{c}$$

Conversely, if

(15) $$\gamma_{i+1}^{c}\leq \gamma_{i}$$

the candidate model is rejected, and the currently deployed model is retained:

(16) $$M_{i+1}=M_{i}$$

Meanwhile, to prevent cumulative degradation caused by successive updates, the system retains the currently deployed model when an update is rejected. In the next cycle, the candidate model is retrained from the initial model $M_{0}$ using the accumulated reliable online samples. Only candidate models that improve performance on the fixed evaluation set are deployed.

## 3. Experimental Results and Discussion

### 3.1. Experimental Setup

**Figure 5:**
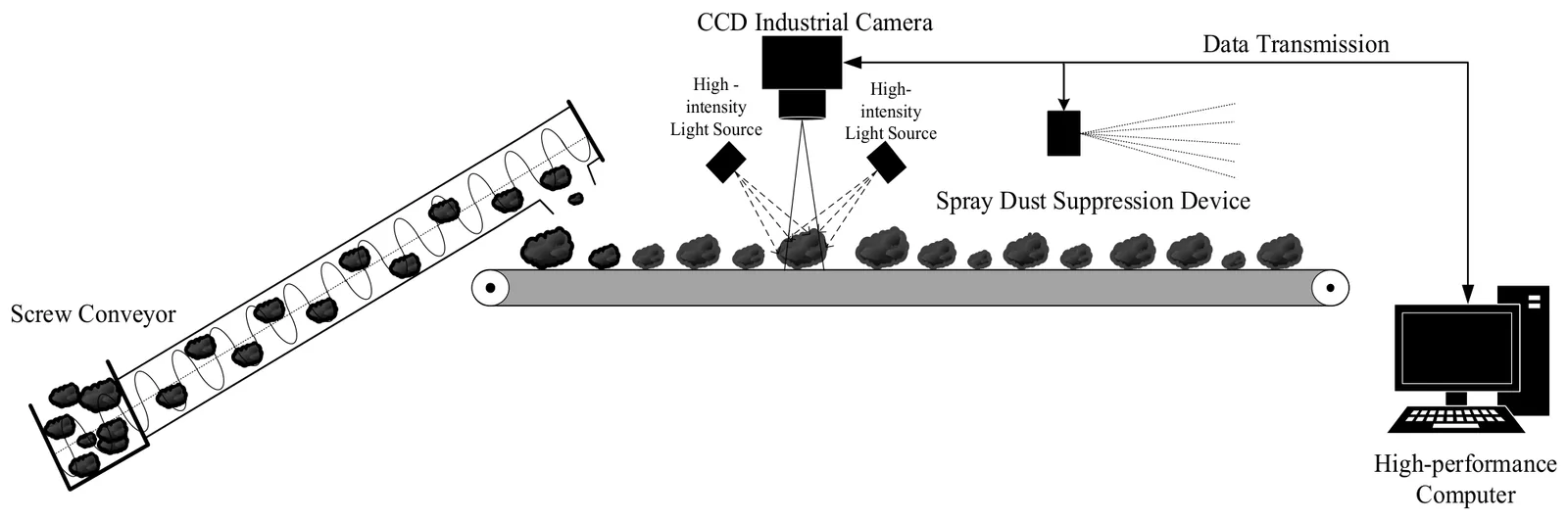
Hardware platform.

The experimental platform consists of a coal conveying system, an image acquisition system, an online detection system, and a model training system, as shown in Figure 5. The hardware system mainly comprises a screw feeder, a conveyor belt, a CCD industrial camera, a white annular industrial light source, a programmable logic controller (PLC), and a spray dust-suppression device. The screw feeder distributes coal fragments uniformly over the surface of the conveyor belt. With the assistance of the white annular industrial light source, the CCD industrial camera captures coal images in real time and transmits the image data to the computing unit. When coal fragments are detected, the computing unit can trigger the PLC to perform subsequent operations such as spray dust suppression.

Five conveyor-belt speed conditions—5×, 10×, 15×, 20×, and 25×—were used in the experiments, corresponding to actual physical speeds of 0.5, 1.0, 1.5, 2.0, and 2.5 m/s, respectively. The image acquisition resolution was 1280 × 960 pixels. A white annular industrial light source with a color temperature of 4000 K was used for illumination, and its brightness was maintained at a constant level. The experimental computing platform was equipped with an Intel^®^ Core™ i5-14600KF CPU operating at 3.50 GHz, with 16 GB of memory, and an NVIDIA GeForce RTX 5060 Ti graphics card with 8 GB of memory. The operating system was Windows 10 x64.

The main hyperparameters used during baseline-model training are presented in Table 6. The number of training epochs was set to 100, and the initial learning rate was set to 0.01. During the online batch-update stage, the candidate model was trained for 30 epochs with a batch size of 20 to reduce the computational-resource requirements of model updating. In the experiments, each epoch required approximately 68 s; therefore, one online batch-training cycle required approximately 34 min. During training, the system used approximately 10 GB of system memory and 6 GB of graphics memory.

To ensure real-time performance during online deployment, a multithreaded deployment scheme was adopted, in which the detection and training threads operated in parallel. The industrial camera acquired conveyor-belt images at 50 fps, corresponding to a 20 ms acquisition interval, and the acquired frames were directly input to the detector at the same rate of 50 Hz, without frame dropping, interpolation, or resampling. The detection thread continuously performed coal-object inference on the incoming image stream, while the training thread was activated only when the batch-update conditions were satisfied. The two threads operated independently, so model training and updating did not interrupt online detection. The experimental results showed that, even when the training thread was running, the inference time was approximately 3 ms per image, which is substantially shorter than the 20 ms acquisition interval. Therefore, the proposed deployment scheme satisfies the real-time requirements of online production-line detection and enables batch model training and updating without interrupting online detection.

Using the 5× speed condition as the baseline, experiments were conducted under five speed conditions: 5×, 10×, 15×, 20×, and 25×. For each speed condition, a validation set containing 100 manually annotated images was constructed, and the class composition was kept consistent across all speed-specific validation sets. To ensure the validity of the evaluation, the images in each validation set did not overlap with either the online sample pool or the candidate-model training data. Recall, mAP50, and mAP50–95 were used as the evaluation metrics, where mAP50–95 is defined as follows:

(17) $$\mathrm{mAP}50–95=\sum_{\mathrm{IoU}=0.5}^{0.95}{\mathrm{mAP}}_{\mathrm{IoU}}$$

In the above equation, mAP50–95 is the average mAP over IoU thresholds ranging from 0.5 to 0.95 in increments of 0.05. It reflects the robustness of the model under bounding-box matching criteria of varying strictness.

### 3.2. Modular Ablation Experiments

To evaluate the sensitivity of the CIW classification thresholds, multiple combinations of lower and upper thresholds were tested, and their Recall, mAP50, and mAP50–95 values were compared under identical experimental conditions. Different threshold combinations alter the trade-off between recall capability and localization accuracy. The threshold combination of (0.3, 0.5) achieved the highest Recall and mAP50. Although its mAP50–95 was not the highest, this study focuses more strongly on suppressing missed detections under high-speed operating conditions. Therefore, 0.3 and 0.5 were selected as the lower and upper CIW classification thresholds, respectively. The detailed results are presented in Table 7.

To verify the effectiveness of the core components of the proposed OIL framework, ablation experiments were conducted using the same initial model, online data, training parameters, and fixed model-update evaluation set. YOLOv8 was used as the base detector, and the Global Attention Mechanism (GAM), the CIW-guided three-level sample management strategy, and the model update and rollback mechanism based on the comprehensive performance metric γ were introduced sequentially.

The CIW-guided three-level sample management strategy includes using the CIW to evaluate the reliability of online detection results and classifying the samples as positive, ambiguous, or negative. Positive samples are used for batch training in the current cycle, ambiguous samples are temporarily stored and re-evaluated after subsequent model updates, and negative samples do not participate in the current training cycle. It should be noted that the CIW does not directly participate in the calculation of the YOLOv8 classification loss, bounding-box regression loss, or total loss. Instead, it is used only for online sample screening and training-data construction. All methods used the same 5× baseline model as the initial model and the same 20× online samples for batch training. Both the candidate and currently deployed models were tested on the fixed multi-speed model-update evaluation set using mAP50, mAP50–95, and Recall as the evaluation metrics. The detailed results are presented in Table 8. The symbols used in Table 8 are defined as follows: √ indicates that the corresponding module is included, whereas x indicates that the corresponding module is not included.

To evaluate the stability of the model training results, ten repeated training runs were conducted while maintaining the same dataset partition, model architecture, and training parameters. The Recall obtained from each run was recorded. Differences among the experimental results were mainly caused by random model-parameter initialization and randomness during the training process. The Recall values obtained from the ten training runs were as follows:

0.641, 0.644, 0.644, 0.642, 0.642, 0.643, 0.643, 0.645, 0.643, 0.643

The mean Recall was 0.643. The standard deviation was calculated as follows:

(18) $$s=\sqrt{\frac{1}{n-1}\sum_{i=1}^{n}{\left(x_{i}-\bar{x}\right)}^{2}}$$

where s denotes the sample standard deviation, which measures the dispersion of the results from repeated experiments relative to their mean; *n* denotes the number of experiments, with n=10 in this study; *i* denotes the *i*-th Recall value; $\bar{x}$ denotes the mean Recall of the ten experiments; and $\left(x_{i}-\bar{x}\right)$ denotes the deviation of an individual experimental result from the mean. Squaring and summing the deviations prevents positive and negative deviations from canceling each other. Dividing the result by n−1 provides an unbiased estimate of the sample variance. Finally, taking the square root ensures that the standard deviation has the same scale as the original Recall metric.

The calculated result was:

s=0.0012

The small standard deviation indicates that the results of the ten experiments exhibited limited variation and that the model demonstrated good stability across different training runs. Because this study considers a single-class object detection task, the number of target classes is limited, and the complexity of the sample distribution is relatively low. The model can therefore converge rapidly and reach a stable state during training, resulting in only minor differences among the final Recall values. Based on the above analysis, although the variance or standard deviation can reflect the stability of the model results, it provides limited discrimination for the proposed method. Therefore, Recall, Precision, mAP, and other detection performance metrics are used to evaluate the model comprehensively.

### 3.3. Experiments Under Different Speed Conditions

To further verify the effectiveness and generalization capability of the OIL closed-loop periodic batch adaptation method under different object-motion speeds, comparative ablation experiments were conducted at multiple speed factors. YOLOv8 and YOLOv8 + GAM, which incorporates the Global Attention Mechanism (GAM), were used as the baseline models. OIL was then integrated with these models to construct YOLOv8 + OIL and YOLOv8 + GAM + OIL. All experiments were conducted using identical training and testing settings, with Recall, mAP50, and mAP50–95 as the evaluation metrics. The detection results of the different models under different speed conditions are presented in Table 9.

As shown in Table 9, the introduction of OIL improved the overall detection performance of YOLOv8 under different speed conditions, with more pronounced gains in high-speed scenarios. At the 25× speed, the Recall, mAP50, and mAP50–95 of YOLOv8 were 0.257, 0.629, and 0.397, respectively. After introducing OIL, the corresponding metrics of YOLOv8 + OIL increased to 0.471, 0.727, and 0.449, corresponding to increases of 21.4, 9.8, and 5.2 percentage points, respectively, compared with the original YOLOv8. These results demonstrate that, as the object motion speed increases and image features become more variable, the closed-loop periodic batch adaptation method can effectively utilize newly acquired samples to continuously optimize the model, thereby improving its adaptability and detection stability under variable-speed conditions.

OIL also demonstrated good generalization capability within the YOLOv8 + GAM framework. Introducing GAM alone improved the feature representation capability of the model to a certain extent under low-speed conditions. For example, at the 5× speed, the Recall and mAP50 increased from 0.765 and 0.872 for YOLOv8 to 0.809 and 0.884 for YOLOv8 + GAM, respectively, corresponding to increases of 4.4 and 1.2 percentage points. However, as the speed factor increased, the adaptability of GAM to high-speed dynamic scenarios became limited. In particular, at the 20× speed, the Recall, mAP50, and mAP50–95 of YOLOv8 + GAM decreased by 7.2, 4.7, and 5.4 percentage points, respectively, compared with YOLOv8. By contrast, YOLOv8 + GAM + OIL achieved better overall performance under high-speed conditions. At the 25× speed, its Recall, mAP50, and mAP50–95 reached 0.643, 0.808, and 0.471, respectively, corresponding to increases of 34.3, 19.2, and 10.3 percentage points, respectively, over YOLOv8 + GAM. These results indicate that OIL not only improves the detection performance of the original YOLOv8 model under variable-speed conditions, but also effectively compensates for the performance degradation of the GAM-enhanced model under high-speed conditions, demonstrating strong adaptability and generalization capability.

**Figure 6:**
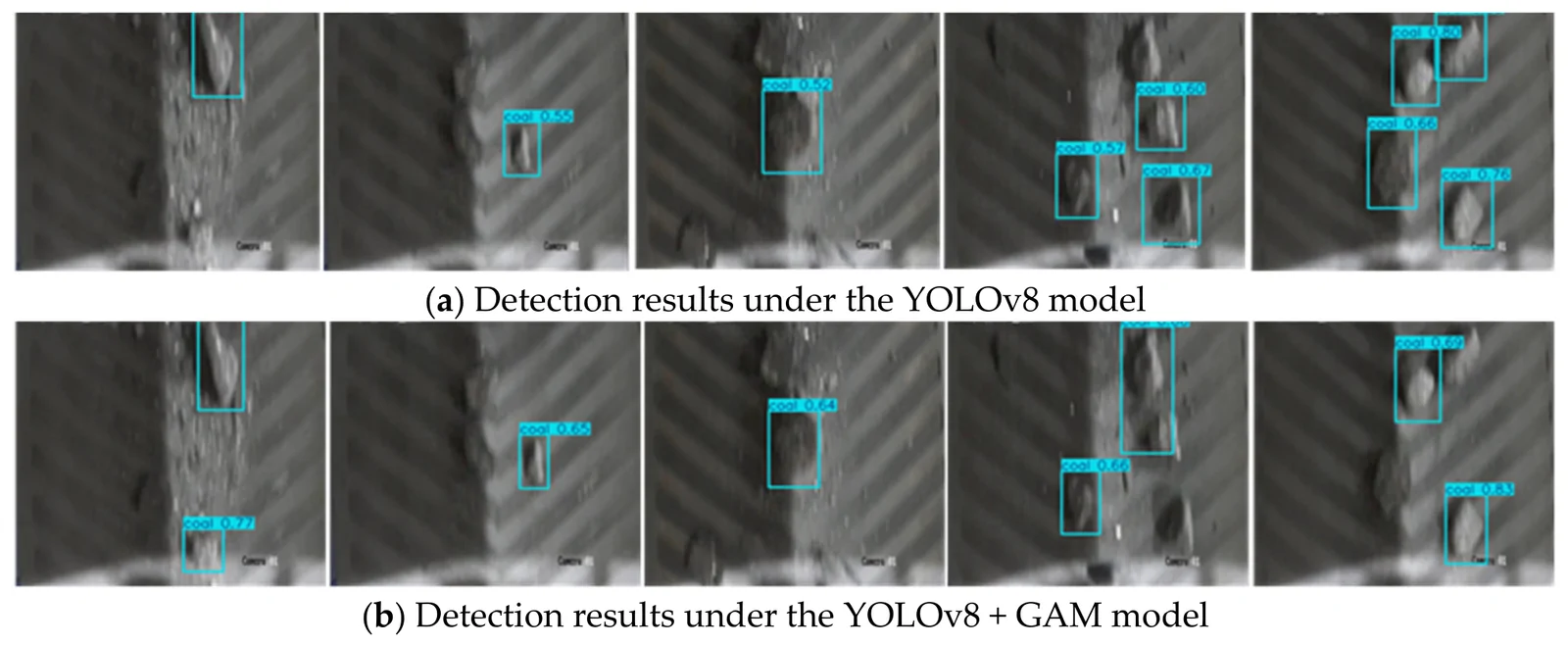

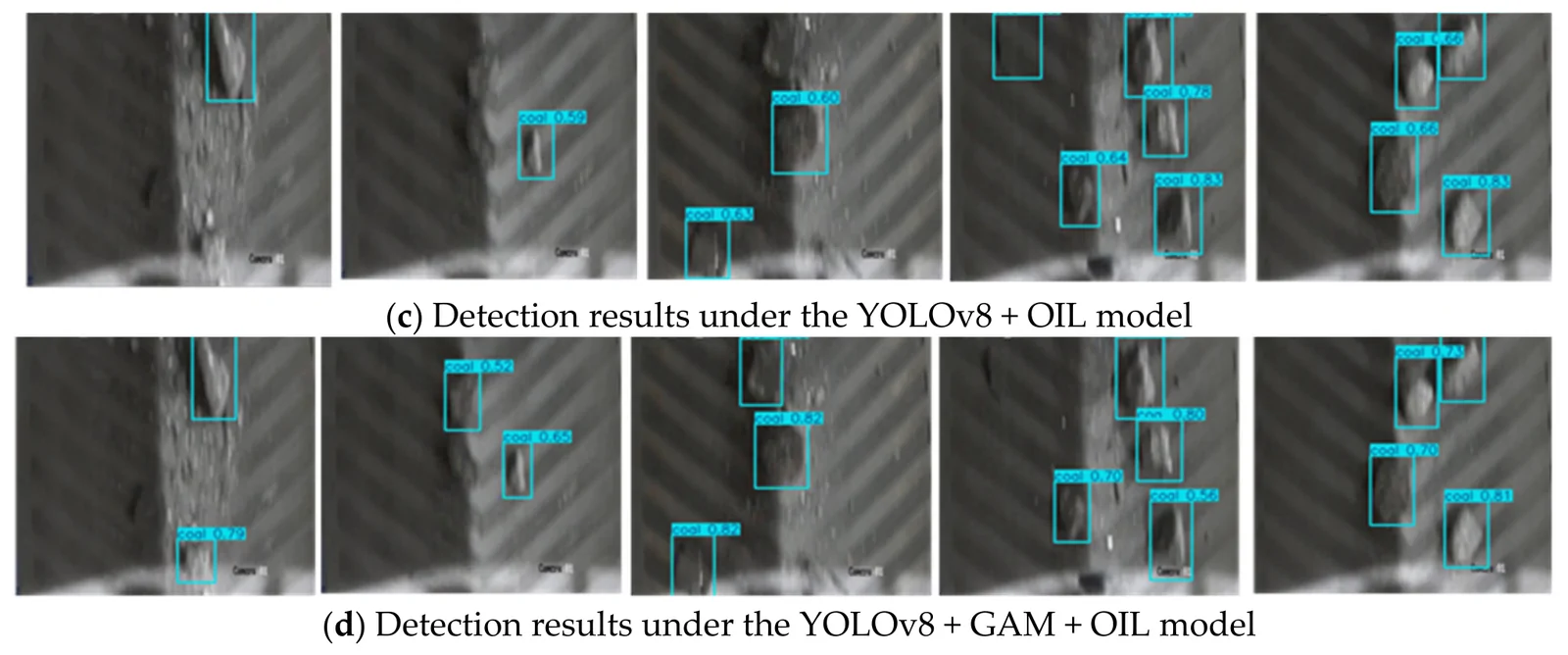
Coal detection results of different models under the 25× speed condition.

The visual detection results of the closed-loop periodic batch adaptation method under high-speed operating conditions are presented in Figure 6. The figure shows the coal-flow detection results obtained using YOLOv8, YOLOv8 + GAM, YOLOv8 + OIL, and YOLOv8 + GAM + OIL at the 25× speed. YOLOv8 serves as the baseline model, whereas YOLOv8 + GAM is used to evaluate the enhancement in feature representation provided by the GAM attention mechanism. YOLOv8 + OIL is used to analyze the improvement in online model adaptability provided by the closed-loop periodic batch adaptation method, and YOLOv8 + GAM + OIL represents the complete proposed model and is used to verify the comprehensive detection performance achieved by combining the two key modules. This ablation design enables the individual contributions of the GAM module, the OIL method, and their combination to high-speed coal-flow detection to be analyzed.

As shown in Figure 6, under the 25× high-speed condition, the original YOLOv8 model produced relatively low detection confidence for some coal objects and exhibited a certain degree of missed detection. After GAM was introduced, the object localization capability of the model improved for some samples; however, its detection stability remained limited in scenarios involving object blur and feature degradation caused by high-speed motion. By contrast, YOLOv8 + OIL enhanced the model’s adaptability to samples collected under different speed conditions through the closed-loop periodic batch adaptation framework, thereby improving the detection confidence and recall performance for some objects. After GAM and OIL were further combined, YOLOv8 + GAM + OIL achieved better performance in terms of object localization completeness, detection confidence, and small-object recognition. These results demonstrate that the proposed closed-loop periodic batch adaptation framework can effectively compensate for feature variations under high-speed operating conditions and improve the detection accuracy and robustness of the model in complex dynamic scenarios.

### 3.4. Discussion

In practical industrial production, the operating speed generally increases gradually from 5× to 25×. As the speed increases, motion blur and changes in the feature distribution lead to a decline in detection performance. Although closed-loop periodic batch adaptation enables the model to adapt to new speed conditions, directly updating the model after each training cycle may cause performance degradation owing to overfitting or error accumulation. Therefore, a rollback strategy is introduced to ensure the stability of model updating.

Under the strategy without rollback, the model is directly deployed after each batch-training cycle. Under the strategy with rollback, an update is performed only when the new model outperforms the current model; otherwise, the original model is retained. The Recall results obtained using the different strategies are presented in Table 10.

As shown in Table 10, when the speed increased from 5× to 25×, Recall decreased from 0.809 to 0.571 without the rollback strategy, indicating that direct model updating may lead to continuous performance degradation. After the rollback strategy was introduced, Recall remained stable at 0.643 from the 15× to the 25× speed condition, preventing a further decline in Recall. These results demonstrate that the rollback strategy can reject ineffective updates and improve the stability of the model update process.

**Figure 7:**
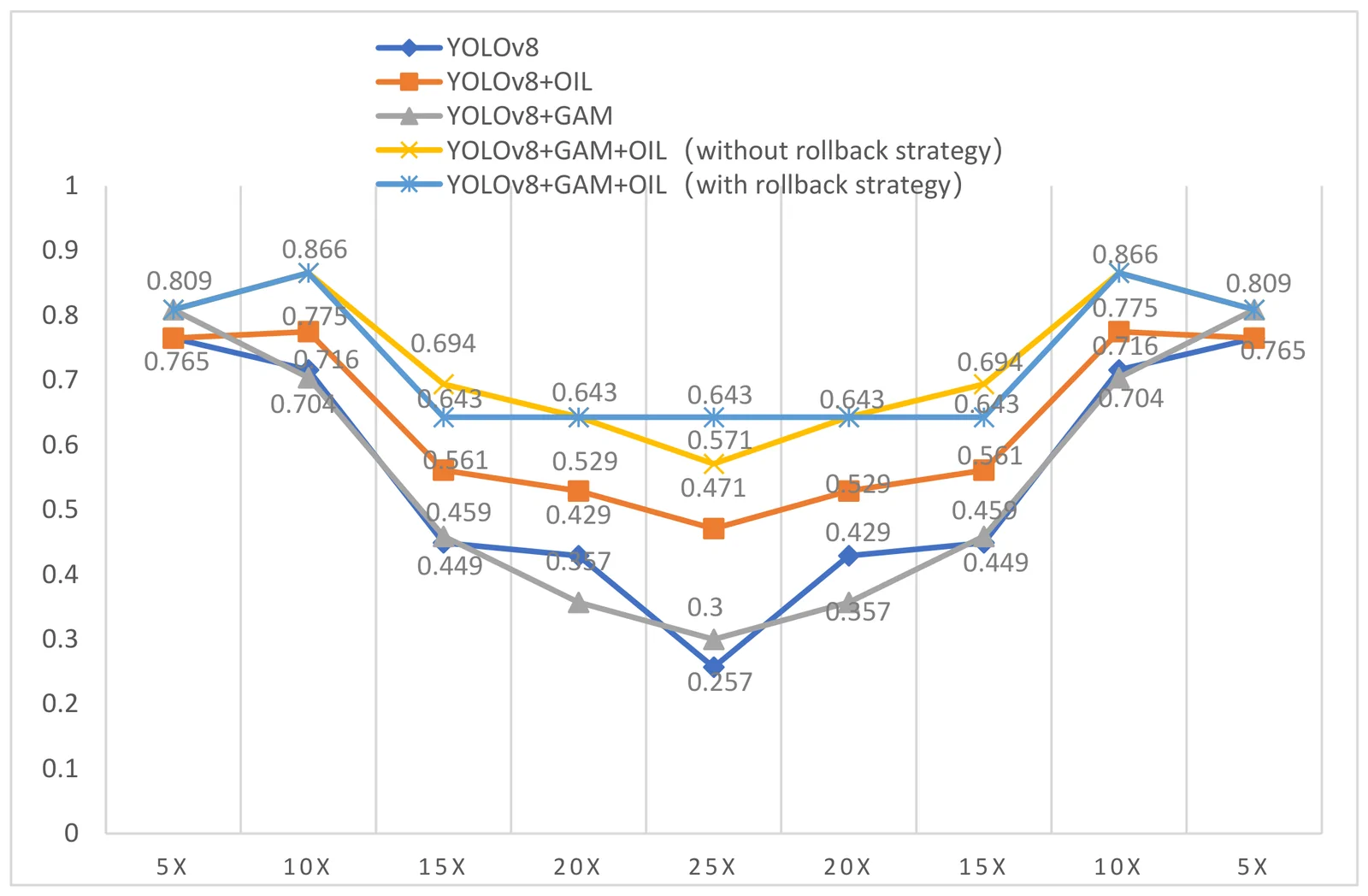
Comparison of Recall Rates for Coal Detection at Different Speeds.

The Recall trends of the different models under dynamic speed variations are shown in Figure 7.

As shown in Figure 7, the Recall values of YOLOv8 and YOLOv8 + GAM decreased substantially as the speed increased. After OIL was introduced, the adaptability of the models to speed variations improved. However, performance degradation still occurred at high speeds without the rollback strategy, and excessive training also caused model overfitting. By contrast, YOLOv8 + GAM + OIL with the rollback strategy maintained more stable Recall as the speed increased from 5× to 25×. Therefore, the rollback strategy can effectively prevent a continuous decline in Recall and improve the reliability of closed-loop periodic batch adaptation in practical deployment.

**Figure 8:**
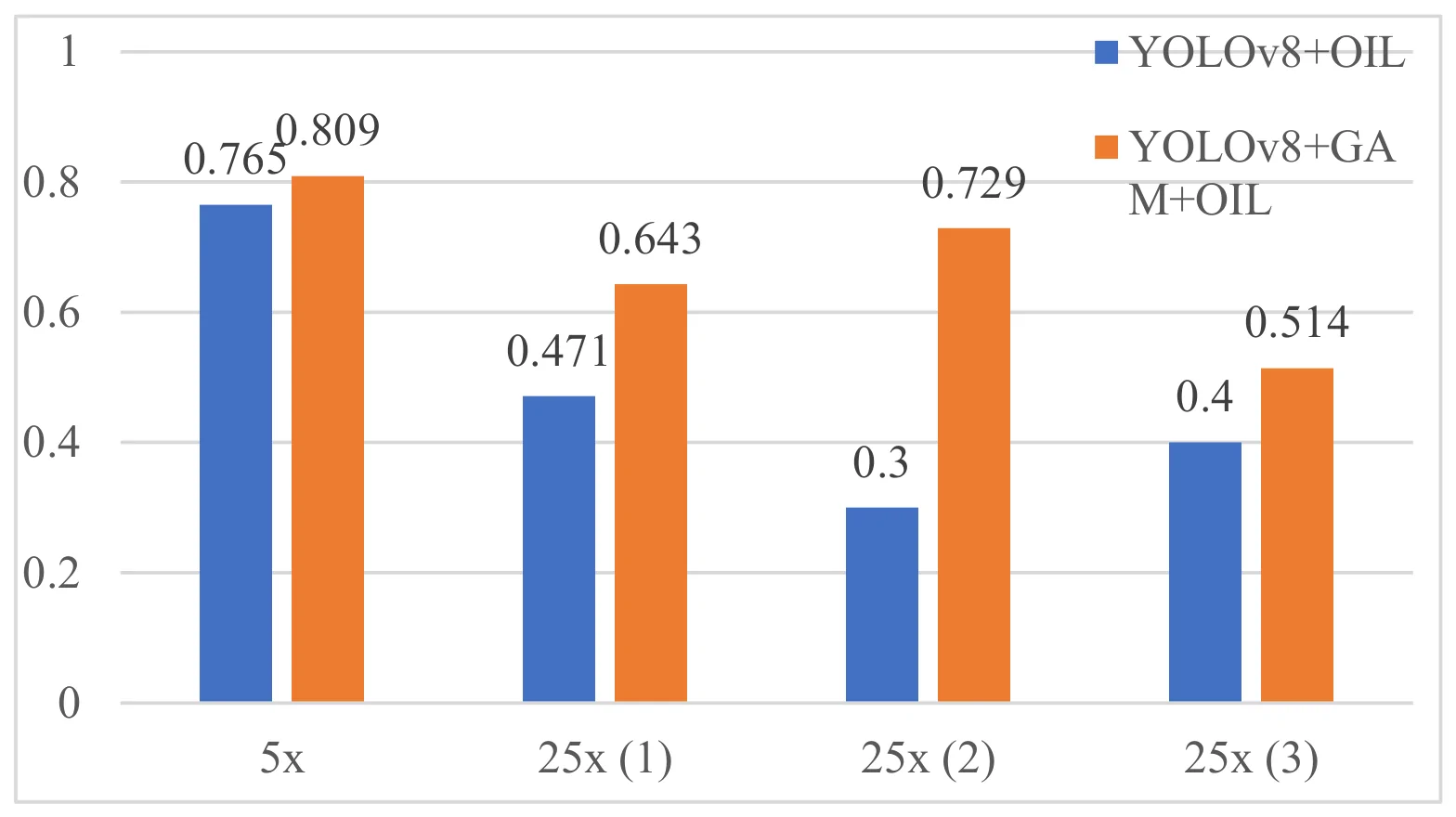
Overfitting Recall Rate Test.

To further investigate the overfitting observed in the absence of the rollback mechanism, a long-term operating condition with a fixed conveyor speed was established. When the conveyor speed remained constant, the proposed method maintained stable performance. However, continuous online updating inherently involves a risk of overfitting. Figure 8 shows the Recall values obtained after five training cycles at the 25× speed.

As shown in Figure 8, the YOLOv8 + OIL model achieved its optimal performance after the first batch-learning cycle, whereas the YOLOv8 + GAM + OIL model achieved its best detection result after the second cycle. As the number of batch-learning cycles continued to increase, the model performance did not improve further and instead exhibited possible overfitting while incurring additional computational overhead. These results indicate that OIL does not require continuous batch training; instead, its training strategy should be dynamically adjusted according to changes in model performance. Therefore, in practical engineering applications, when the conveyor speed remains stable and recall no longer improves, the current batch-learning process should be terminated. When necessary, online batch adaptation should be restarted from the initial model state to avoid overfitting and reduce computational-resource consumption.

In addition, because video-segment-level partitioning was not adopted, potential temporal correlations may still exist among frames extracted from continuous video streams, and visually similar adjacent frames may appear across different data subsets. Frame-interval sampling, in which one image is selected every N frames according to the video frame rate, object motion speed, and required sample diversity, could reduce redundancy among adjacent frames and lower the probability of highly similar frames appearing in different subsets. However, in a closed-loop periodic batch adaptation framework, a larger sampling interval may increase the time required to accumulate sufficient samples for triggering model updates, thereby weakening the timeliness of adaptation to new operating conditions. For this reason, frame-interval sampling was not implemented in the current framework.

## 4. Conclusions

The proposed method is a high-accuracy closed-loop periodic batch adaptation method for object detection on variable-speed production lines and mitigates the performance degradation caused by variations in conveyor-belt speed. First, an improved YOLOv8-based method is used for object detection, enabling more accurate extraction of object detection features. Second, different confidence intervals are defined to construct new samples for the closed-loop periodic batch adaptation model, effectively addressing the shortage of object detection samples. Finally, a model update strategy is implemented based on detection results and efficiency, enabling rapid model updating and deployment. In experiments involving speed variations from 5× to 25×, the proposed method maintained a Recall above 0.643. At the 25× speed, compared with the YOLOv8 + GAM network, the proposed method increased Recall from 0.300 to 0.643, mAP50 from 0.616 to 0.808, and mAP50–95 from 0.368 to 0.471. The experimental results demonstrate that the proposed method achieves significant performance improvements in variable-speed coal detection scenarios, particularly at the 25× speed. The current experiments remain limited to single-class coal detection and a maximum speed of 25×. Further validation is required under higher speeds, complex illumination, dust, occlusion, and multiclass conditions. Future work will extend the method to lightweight deployment and multi-mode integration and will use more diverse datasets covering dust, different illumination conditions, occlusion, and complex backgrounds to further evaluate the robustness and generalization capability of the framework.

## Figures and Tables

**Table 1:** Compares representative existing methods with the proposed OIL method.

Comparison Dimension	Representative Existing Studies	OIL Method
Detection framework	Static models with fixed parameters after training.	Closed-loop learning comprising data acquisition, training, deployment, and feedback-driven updating.
Attention mechanism	Some studies have introduced GAM, but few have combined it with variable-speed scenarios.	GAM-enhanced YOLOv8 integrates local and global features.
Sample classification strategy	Binary classification into positive and negative samples.	A CIW-based three-level strategy comprising positive, ambiguous, and negative samples.
Data efficiency	Requires a large amount of annotated data.	Efficiently utilizes 1100 images through ambiguous- and negative-sample mining.
Variable-speed adaptability	Generally relies on time-consuming deblurring preprocessing.	Directly adapts to speed variations without additional deblurring.
Model update mechanism	Static deployment; retraining is required in new environments.	Feedback-driven conditional online updating without complete retraining.

**Table 2:** CIW-based sample classification.

Sample Type	Conf Performance	IoU Performance	θ Value
Negative Sample	Low (<0.3)	Low (<0.3)	<0.3
	Low (<0.3)	/	
	/	Low (<0.3)	
Ambiguous Sample	Moderate (0.3–0.5)	Moderate (0.3–0.5)	0.3–0.5
Positive Sample	High (>0.5)	High (>0.5)	>0.5

**Table 3:** Composition of the 5× baseline dataset.

Dataset	Clear Coal Positive Samples	Manually Annotated Blurred Coal Samples	Coal-Free Background Samples	Total
Training Set	500	200	100	800
Validation Set	50	30	20	100
Test Set	100	60	40	200
Total	650	290	160	1100

**Table 4:** Allocation of baseline data and online data for batch training and internal monitoring.

Dataset	Batch Training Set	Internal Batch Monitoring Set
5× Baseline Dataset	800 (Manual Annotations)	200 (Manual Annotations)
New Operating Conditions	200 (Pseudo-Labels)	100 (Pseudo-Labels)
Total	1000	300

**Table 5:** Fixed model-update evaluation set.

Speed Condition	Clear Coal Positive Samples	Manually Annotated Blurred Coal Samples	Coal-Free Background Negative Samples	Total	Primary Purpose
5×	50	30	20	100	Evaluation of model performance at the baseline speed
10×	50	30	20	100	Evaluation of model performance under medium–low-speed conditions
15×	50	30	20	100	Evaluation of model performance under medium-speed conditions
20×	50	30	20	100	Evaluation of model performance under high-speed conditions
25×	50	30	20	100	Evaluation of model performance under high-speed motion-blur conditions
Total	250	150	100	500	Fixed model-update evaluation set

**Table 6:** Candidate hyperparameter values and final selected values.

Parameter	Candidate Values	Final Value	Selection Criterion
Input image size	416, 512, 640, 768	640	Balance between detection accuracy and inference efficiency
Candidate-model training epochs	20, 30, 50	30	Convergence of the training and validation losses
Baseline-model training epochs	50, 100, 150	100	Convergence of the training and validation losses
Initial learning rate	0.001, 0.005, 0.01	0.01	Optimal validation mAP and stable training loss
Confidence threshold	0.3, 0.5, 0.7	0.5	Balance between false and missed detections
Positive-Sample threshold	0.3, 0.5, 0.6	0.5	Ensuring reliable localization of positive samples
Negative-Sample threshold	0.2, 0.3, 0.4	0.3	Filtering unreliable pseudo-labels

**Table 7:** Sensitivity analysis of the CIW thresholds.

Lower Threshold	Upper Threshold	Recall	mAP50	mAP50–95
0.2	0.5	0.863	0.953	0.603
0.3	0.5	0.900	0.976	0.586
0.3	0.4	0.896	0.949	0.519
0.3	0.6	0.879	0.948	0.611
0.4	0.5	0.858	0.932	0.612

**Table 8:** Ablation results for the core modules.

Method	GAM	CIW	γ-Based Updating and Rollback	Recall	mAP50	mAP50–95
YOLOv8	x	x	x	0.429	0.702	0.363
YOLOv8 + GAM	√	x	x	0.357	0.655	0.309
YOLOv8 + GAM + OIL Conf (>0.5)	√	x	√	0.493	0.626	0.422
YOLOv8 + GAM + OIL CIW	√	√	√	0.643	0.766	0.444

**Table 9:** Comparative results of different models under varying speed conditions.

Speed	Recall				mAP50				mAP50–95			
	YOLOv8	YOLOv8 + OIL	YOLOv8 + GAM	YOLOv8 + GAM + OIL	YOLOv8	YOLOv8 + OIL	YOLOv8 + GAM	YOLOv8 + GAM + OIL	YOLOv8	YOLOv8 + OIL	YOLOv8 + GAM	YOLOv8 + GAM + OIL
5×	0.765	0.765	0.809	0.809	0.872	0.872	0.884	0.884	0.610	0.610	0.567	0.567
10×	0.716	0.775	0.704	0.866	0.811	0.861	0.819	0.869	0.521	0.551	0.518	0.553
15×	0.449	0.561	0.459	0.643	0.708	0.737	0.688	0.786	0.459	0.446	0.395	0.469
20×	0.429	0.529	0.357	0.643	0.702	0.758	0.655	0.766	0.363	0.465	0.309	0.444
25×	0.257	0.471	0.300	0.643	0.629	0.727	0.616	0.808	0.397	0.449	0.368	0.471

**Table 10:** Recall comparison with and without the rollback strategy under different speed conditions.

Strategy	5×	10×	15×	20×	25×	20×	15×	10×	5×
Without rollback strategy	0.809	0.866	0.694	0.643	0.571	0.643	0.694	0.866	0.809
With rollback strategy	0.809	0.866	0.643	0.643	0.643	0.643	0.643	0.866	0.809

## Data Availability

The original contributions presented in this study are included in the article. Further inquiries can be directed to the corresponding author.
